# Malaria parasites in macaques in Thailand: stump-tailed macaques (*Macaca arctoides*) are new natural hosts for *Plasmodium knowlesi*, *Plasmodium inui, Plasmodium coatneyi* and *Plasmodium fieldi*

**DOI:** 10.1186/s12936-020-03424-0

**Published:** 2020-10-01

**Authors:** Wirasak Fungfuang, Chanya Udom, Daraka Tongthainan, Khamisah Abdul Kadir, Balbir Singh

**Affiliations:** 1 Department of Zoology, Faculty of Science, Kasetsart University, Bangkok, 10900 Thailand; 2 Faculty of Veterinary Medicine, Kasetsart University, Bangkok, 10900 Thailand; 3 Malaria Research Centre, Universiti Malaysia Sarawak, 94300 Kota Samarahan, Sarawak, Malaysia

**Keywords:** *Plasmodium knowlesi*, *Plasmodium cynomolgi*, *Plasmodium inui*, *Macaca arctoides*, Zoonosis

## Abstract

**Background:**

Certain species of macaques are natural hosts of *Plasmodium knowlesi* and *Plasmodium cynomolgi*, which can both cause malaria in humans, and *Plasmodium inui,* which can be experimentally transmitted to humans. A significant number of zoonotic malaria cases have been reported in humans throughout Southeast Asia, including Thailand. There have been only two studies undertaken in Thailand to identify malaria parasites in non-human primates in 6 provinces. The objective of this study was to determine the prevalence of *P. knowlesi, P. cynomolgi, P. inui, Plasmodium coatneyi* and *Plasmodium fieldi* in non-human primates from 4 new locations in Thailand.

**Methods:**

A total of 93 blood samples from *Macaca fascicularis*, *Macaca leonina* and *Macaca arctoides* were collected from four locations in Thailand: 32 were captive *M. fascicularis* from Chachoengsao Province (CHA), 4 were wild *M. fascicularis* from Ranong Province (RAN), 32 were wild *M. arctoides* from Prachuap Kiri Khan Province (PRA), and 25 were wild *M. leonina* from Nakornratchasima Province (NAK). DNA was extracted from these samples and analysed by nested PCR assays to detect *Plasmodium,* and subsequently to detect *P. knowlesi, P. coatneyi*, *P. cynomolgi, P. inui* and *P. fieldi*.

**Results:**

Twenty-seven of the 93 (29%) samples were *Plasmodium*-positive by nested PCR assays. Among wild macaques, all 4 M*. fascicularis* at RAN were infected with malaria parasites followed by 50% of 32 M*. arctoides* at PRA and 20% of 25 M*. leonina* at NAK. Only 2 (6.3%) of the 32 captive *M. fascicularis* at CHA were malaria-positive. All 5 species of *Plasmodium* were detected and 16 (59.3%) of the 27 macaques had single infections, 9 had double and 2 had triple infections. The composition of *Plasmodium* species in macaques at each sampling site was different. *Macaca arctoides* from PRA were infected with *P. knowlesi, P. coatneyi*, *P. cynomolgi, P. inui* and *P. fieldi*.

**Conclusions:**

The prevalence and species of *Plasmodium* varied among the wild and captive macaques, and between macaques at 4 sampling sites in Thailand. *Macaca arctoides* is a new natural host for *P. knowlesi, P. inui, P. coatneyi* and *P. fieldi.*

## Background

Malaria is caused by the protozoan parasite members of the genus *Plasmodium* and over 250 *Plasmodium* spp. have been described in mammals, birds, rodents and reptiles [1–3]. More than 30 malaria species have been reported in non-human primates and the following have been either naturally acquired or experimentally transmitted to humans by mosquitoes: *Plasmodium cynomolgi*, *Plasmodium knowlesi* and *Plasmodium inui* from Old World monkeys, *Plasmodium brasilianum* and *Plasmodium simium* from New World monkeys and *Plasmodium schwetzi* from chimpanzees [2, 4–7].

*Plasmodium knowlesi* was first reported as a significant cause of human malaria in Malaysia in 2004 [8]. Subsequently, naturally-acquired human infections with *P. knowlesi* were documented in several other countries in Southeast Asia including Thailand [9, 10], Indonesia [11], Philippines [12], Singapore [13], Vietnam [14], Cambodia [15], Laos [16] and Myanmar [17]. Furthermore, travelers have returned to their home countries after visiting Thailand and other Southeast Asian countries with knowlesi malaria [18, 19]. In Thailand, the first locally acquired natural infection with *P. knowlesi* was reported in 2004, in a patient who had visited the forest in Prachuap Kiri Khan Province, Southern Thailand near the Myanmar border [9]. Subsequently, *P. knowlesi* infected patients have been reported in Tak, Chantaburi, Yala, Narathiwat, Prachuap Kiri Khan and Ranong Provinces [20–22]. These areas are located near the borders of Cambodia, Myanmar and Malaysia. Tourists visiting Ranong Province, and South Western Thailand have also returned to their home countries in Germany [23–25] and France [26] with knowlesi malaria. Besides *P. knowlesi*, naturally acquired human *P. cynomolgi* infections have recently been reported in Peninsular Malaysia [27], Malaysian Borneo [28, 29] and Cambodia [30] and in a Danish tourist who had visited Peninsular Malaysia and Thailand [31]. Although naturally-acquired human infections with *P. inui* have not been described, *P. inui* can cause malaria in humans by blood passage [4] or through mosquito bites in the laboratory [32].

The main natural hosts of *P. knowlesi*, *P. cynomolgi* and *P. inui* are long-tailed macaques (*Macaca fascicularis*) and pig-tailed macaques (*Macaca nemestrina*) which are found in nature in Southeast Asia [2]. There have also been reports of a *P. knowlesi* infection in a banded leaf monkey (*Presbytis melalophos*) in Peninsular Malaysia [33] and a dusky leaf monkey (*Semnopithecus obscurus*) in Thailand [34]. The other natural hosts of *P. cynomolgi* are *Macaca radiata, Macaca cyclopis, Macaca sinica, Macaca mulatta, Presbytis cristasus* and *Presbytis entellus* [2]. *Plasmodium inui* also naturally infects many monkey species, including *M. cyclopis, M. mulatta, M. radiata, Presbytis cristasus, P. obscurus* and *Cynopithecus niger* [2]. Besides the natural hosts described above, a number of non-human primate hosts can be experimentally infected with *P. knowlesi* and *P. cynomolgi*. Experimental hosts of *P. knowlesi* described to date have included *Callithrix jacchus*, *Cebus* spp., *Cercocebus fuliginosus*, *C. cephus*, *Cynochepalus papio*, *Hoolock hoolock*, *M. radiata*, *M. arctoides*, *Papio doguera*, *Papio jubilaeus*, *Papio papio*, *Presbytis cristatus*, *Saimiri sciureus* and *Semnopithecus entellus* [2]. For *P. cynomolgi* experimental hosts have included *M. mulatta*, *Cercopithecus aethiops*, *Cebus capucinus* and *Papio papio* [2].

In Thailand, six species of macaques have been identified in nature; *M. fascicularis*, *M. nemestrina*, the northern pig-tailed macaque (*M. leonina*), the rhesus macaque (*M. mulatta*), the stump-tailed macaque (*M. arctoides*) and the Assamese macaque (*Macaca assamensis*) [35–39]. There have been only two studies undertaken to determine the prevalence of malaria in non-human primates in Thailand. Using molecular techniques, *P. inui and P. coatneyi* were identified in wild-caught *M. fascicularis* in Ranong Province, near the Myanmar border [36]. Then in 2010, *P. inui, P. coatneyi* and *P. knowlesi* were described in wild-caught *M. fascicularis* and *M. nemestrina* in Yala and Narathiwat Provinces, in Southern Thailand near the Malaysian border [34]. It is important to determine the geographical range of malaria-positive non-human primates to inform the public of the risks of acquiring zoonotic malaria. The objective of this study was to determine the prevalence of malaria parasites in non-human primates from 4 new locations in Thailand.

## Methods

### Collection of samples

**Fig. 1:**
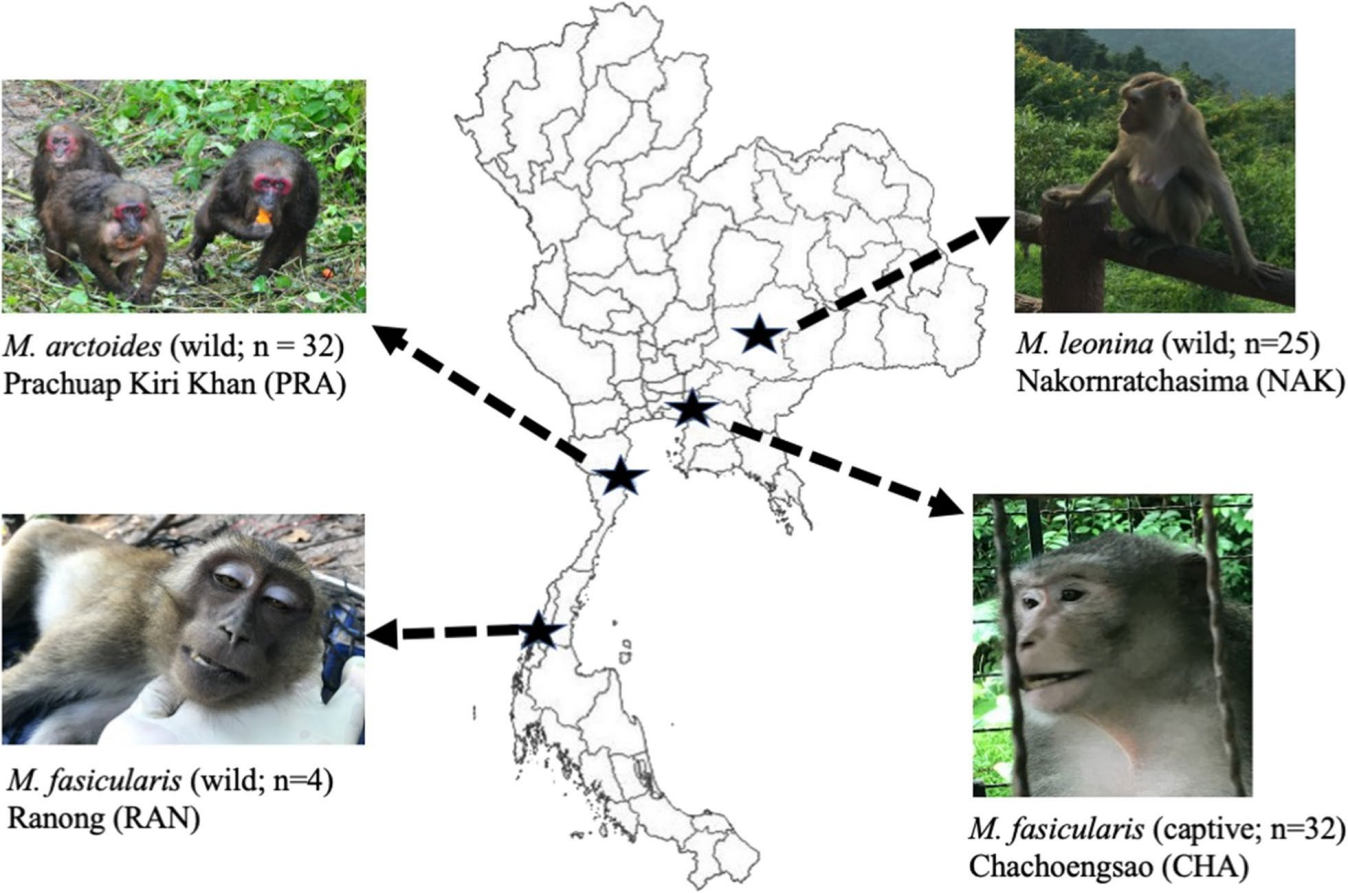
Map of Thailand showing sampling sites and the species of macaques sampled. CHA, Chachoengsao Province; NAK, Nakornratchasima Province; RAN, Ranong Province; PRA, Prachuap Kiri Khan Province

A total of 93 blood samples were collected from captive and wild macaques in 4 areas of Thailand from 2017–July 2019 (Fig. 1). These included 32 samples from captive *M. fascicularis* at the Krabok Koo Wildlife Breeding Center, Chachoengsao Province (CHA), 32 samples from wild *M. arctoides* at the Pa La U waterfall, Huahin district, Prachuap Kiri Khan Province (PRA), 25 samples from wild *M. leonina* at the Khao Yai National Park, Nakornratchasima Province (NAK) and 4 samples from wild *M. fascicularis* at Chang Island, Mu Ko Ranong National Park, Ranong Province (RAN). The Department of National Parks, Wildlife and Plant Conservation, Thailand, approved the protocol for study in a protected area, collection of the blood samples and released of wild macaques (Permit Number: 0909.204/14187). All procedures were carried out in accordance with the Guide for the Care and Use of Laboratory Animals of the National Institutes of Health, U.S.A., and were approved by the Animal use and care committee of Kasetsart University Research and Development Institute, Kasetsart University, Thailand (ID: ACKU59-SCI-011).

The macaques were trapped and anesthetized intramuscularly with Tiletamine-Zolazepam (2–5 mg/kg body weight) and Xylazine hydrochloride (0.5–2 mg/kg body weight). The blood samples (maximum of 3 ml per animal) were collected using a syringe from the femoral vein of the macaques into a tube with EDTA. From the EDTA tubes, three 40–50 μL of blood from each sample were transferred to Whatman 3 MM filter paper. The samples were initially kept at room temperature and were transported to the Department of Zoology, Faculty of Science, Kasetsart University until molecular analysis. Blood spots on filter papers were transported to the Malaria Research Centre, Universiti Malaysia Sarawak, Kota Samarahan, Sarawak, Malaysia for DNA extraction and molecular analysis.

### Analysis of samples

DNA was extracted from blood spots on filter papers with the use of InstaGene (Bio-Rad Laboratories, USA) as described previously [40]. The DNA samples were first examined by nested PCR assays based on the small subunit ribosomal RNA genes of *Plasmodium* with the aid of genus-specific primers (rPLU1 and rPLU5 in nest 1 amplification, and rPLU3 and rPLU4 in nest 2) as described previously [41]. *Plasmodium*-positive samples were then examined by nested PCR assays using species-specific primers to detect *P. knowlesi, P. coatneyi*, *P. cynomolgi, P. inui* and *P. fieldi,* as described previously [42]. The products of the PCR amplification were analysed by gel electrophoresis in 2.7% agarose gels and were stained by Sybersafe before being observed under UV light.

### Statistical analysis

The Fisher-Freeman-Halton exact test was used to compute the exact probabilities of the prevalence of *Plasmodium* species between locations and among troops of macaques. The statistical analysis was undertaken using software R (version 3.5.2) and statistical significance was set at P < 0.05.

## Results

Out of 93 macaque blood samples examined by nested PCR assays, 27 (29%) were *Plasmodium*-positive (Table 1). Among wild macaques, all 4 M*. fascicularis* at RAN were infected with malaria parasites followed by 50% of *M. arctoides* at PRA and 20% *M. leonina* at NAK. Only 6.3% of the captive *M. fascicularis* at CHA were malaria-positive.

**Table 1:** Malaria parasites identified in macaques by location

Infection type	*Plasmodium* species	Number of macaques infected at each location				
		CHA	NAK	RAN	PRA	Total positive
		*M. fascicularis* (captive; n = 32)	*M. leonina* (wild; n = 25)	*M. fascicularis* (wild; n = 4)	*M. arctoides* (wild; n = 32)	
Single	Pk				1	1
	Pin		1	3	3	7
	Pcy	2	1		1	4
	Pct				1	1
	Pfld				3	3
Double	Pin, Pcy				1	1
	Pin, Pfld		3	1		4
	Pcy, Pct				1	1
	Pct, Pfld				3	3
Triple	Pin, Pcy, Pfld				2	2
Total *Plasmodium*-positive		2	5	4	16	27
Percentage *Plasmodium*-positive		6.25	20	100	50	

CHA, Chachoengsao Province; NAK, Nakornratchasima Province; RAN, Ranong Province; PRA, Prachuap Kiri Khan Province; Pk, *P. knowlesi*; Pin, *P. inui*; Pcy, *P. cynomolgi*; Pct, *P. coatneyi*; Pfld, *P. fieldi*

All five species of *Plasmodium* parasites were detected; *P. knowlesi*, *P. inui*, *P. cynomolgi*, *P. coatneyi* and *P. fieldi*. The majority of malaria-positive macaques (59.3%) had mono-infections, 33.3% had double and 7.4% had triple infection of *Plasmodium* spp. Overall, *P. inui* was the most prevalent species detected, occurring in 35% of the malaria-positive macaques, followed by *P. fieldi* (30%), *P. cynomolgi* (20%), *P. coatneyi* (12.5%) and *P. knowlesi* (2.5%).

The prevalence of each parasite species varied significantly among the macaque species and sites of collection (Table 1). The *P* value for the Fisher exact test was 10^–5^ indicating that the prevalence of each species of *Plasmodium* species exhibited bias among different locations. The prevalence of each *Plasmodium* species infection in *M. arctoides* was higher than that for the other macaques (P = 0.007). and all five species of *Plasmodium* that were tested for, were detected in these wild *M. arctoides*.

## Discussion

The prevalence of *Plasmodium* spp. in captive *M. fascicularis* at the Krabok Koo Wildlife breeding centre at CHA was much lower than that observed in wild macaques from the other 3 provinces. These macaques were reported to be trapped and brought to the centre due to human-monkey conflict. It is unlikely that these *M. fascicularis* acquired their infections at the breeding centre because if there were vectors of malaria in the vicinity and active transmission of malaria, the prevalence of malaria parasites would have been much higher at this location. A more likely explanation is that the 2 M*. fascicularis* that were infected had acquired their infections before they were transported to the breeding center. Among the infection rates in wild macaques, all the *M. fascicularis* studied from RAN had malaria parasites, while half the 32 wild *M. arctoides* studied at PRA and a quarter of the 25 M*. leonina* at NAK were infected. However, the current study is a relatively small one where only 4 M*. fascicularis* at RAN were examined, and future surveillance involving a larger number of samples is needed to determine accurately the prevalence of malaria infection among the wild macaques residing in all these areas. In future studies it would also be preferable to sample wild macaques rather than captive monkeys since this would provide more accurate information on zoonotic malaria parasites that are present in that particular ecosystem and could pose a threat to humans visiting these areas for recreation or hunting.

A complex nature of *Plasmodium* spp. infections was observed among the wild macaques studied, which is similar to the results of previous studies on wild macaques in Peninsular Malaysia, Malaysian Borneo, Thailand, Singapore, Laos, Cambodia and the Philippines [34, 42–46]. The DNA samples were each collected at single time points and the PCR results may not reflect the actual prevalence or the total number of species of *Plasmodium* present in each host since it has been observed that the parasitaemia of malaria parasites fluctuate over time within a host [2]. Furthermore, late asexual stage parasites of *P. coatneyi* sequester, so in a synchronous infection the parasites would only be detected in the blood if the majority of the parasites were at the early trophozoite or ring stage during sample collection [2]. The presence of multiple infections leads to difficulties in accurate identification by microscopy, so molecular methods are essential for identification of the various species of *Plasmodium* spp. in macaques and other primates.

In the current study, *P. inui* was the most prevalent *Plasmodium* spp. parasite detected in *M. leonina*, similar with the previous studies conducted on wild *M. fascicularis* and *M. nemestrina* in Thailand [34], Malaysian Borneo [42], and Peninsular Malaysia [44]. However, the composition of *Plasmodium* species within macaques at each sampling site was different, and this was also observed in the study on *M. fascicularis* in the Philippines [45] and in the study by Zhang et al*.* of regional populations of *M. fascicularis* across Southeast Asia [46]. The reasons for differences in the number of animals infected with multiple malaria parasites at each site are multifactorial, and probably include differences in host genetics and susceptibility, single time point sampling and differences in the vectors between sites [2].

*Macaca arctoides,* or stump-tailed macaques, are found in forested areas in continental Southeast Asian countries including Myanmar, Thailand, Vietnam, Laos, Peninsular Malaysia, and in south-western China and north-eastern India [35, 38]. In Thailand, the populations are distributed in the south in peninsular Thailand, and in central and north-western Thailand, mainly in the forests associated with limestone [35, 38]. *Macaca arctoides* are natural hosts for *P. cynomolgi* and have been shown experimentally to be susceptible to infection by *P. knowlesi* and *P. coatneyi* [2]. In a previous study, no malaria parasites were found in 4 M*. arctoides* from RAN in southern Thailand [36]. In the current study, *M. arctoides* at PRA were infected with *P. knowlesi, P. coatneyi*, *P. cynomolgi, P. inui* and *P. fieldi.* Since *M. arctoides* have previously been reported as natural hosts for *P. cynomolgi* [2], this is the first documentation of natural infections of *M. arctoides* with *P. knowlesi, P. coatneyi*, *P. inui* and *P. fieldi.*

Macaques from the following six provinces in Thailand had previously been trapped and studied for malaria parasites by Seethamchai et al. in 2006 [36] and by Putaporntip et al. in 2010; RAN, PRA, Pathalung, Pattani, Yala and Narathiwat [34]. In the current study, macaque samples from CHA and NAK, two provinces which had not been studied previously, were examined by molecular detection assays for malaria parasites. For the other two provinces (RAN and PRA), sampling was undertaken at sites which differed from the previous study in 2006. The macaque collecting site at RAN in the current study was Chang island in the Andaman sea, whereas Seethamchai et al. studied *M. fascicularis* from the mainland [36]. For PRA, these workers examined semi-wild *M. fascicularis* at the Wat Khao Takieb temple, while the current study focused on wild *M. arctoides* at the Pa La U waterfall in Huahin District, 76 km away from the Wat Khao Takieb temple. Therefore, a total of 8 different sites from 6 of the 77 provinces in Thailand have been studied so far to determine the prevalence of malaria parasites in macaques. Further studies, utilizing molecular detection assays and involving more sampling sites and a larger number of monkey blood samples per study site are necessary to determine the geographical range of macaques infected with zoonotic malaria parasites in Thailand and also in other countries in Southeast Asia.

## Conclusions

Macaques sampled from all 4 locations in Thailand were infected with malaria parasites. The prevalence of malaria parasites varied among the species of monkeys and the sites of sample collection. This is the first report of natural infections of *M. arctoides* with *P. knowlesi, P. coatneyi*, *P. inui* and *P. fieldi.* The presence of macaques infected with malaria parasites, some that are transmissible to humans, presents a potential public health risk to the local population.

## Data Availability

Not applicable.

## Abbreviations

CHA: Chachoengsao Province
RAN: Ranong Province
PRA: Prachuap Kiri Khan Province
NAK: Nakornratchasima Province
Pk: *P. knowlesi*; Pin: *P. inui*
Pcy: *P. cynomolgi*; Pct: *P. coatneyi*; Pfld: *P. fieldi*
